# Outcomes of patients with amyotrophic lateral sclerosis admitted to the ICU for acute respiratory failure

**DOI:** 10.1016/j.aicoj.2026.100183

**Published:** 2026-10-12

**Authors:** Adam Celier, Aymeric Lanore, Adele Hesters, Sarah Hussein, Raouf Benchikh, Gaelle Bruneteau, Capucine Morélot-Panzini, Mélodie Parfait, Mathilde Piljan, Loic Le Guennec, Nicolas Weiss, Sophie Demeret, Julien Mayaux, Clemence Marois

**Affiliations:** a AP-HP, Groupe Hospitalier Universitaire APHP-Sorbonne Université, site Pitié-Salpêtrière, Service de Médecine Intensive et Réanimation à orientation neurologique, Paris, France; b Sorbonne Université, Institut du Cerveau - Paris Brain Institute - ICM, Inserm, CNRS, Paris, France; c AP-HP, Groupe Hospitalier Universitaire APHP-Sorbonne Université, site Pitié-Salpêtrière, Département de neurologie, Centre d’Investigation Clinique Neurosciences, Hôpital Pitié-Salpêtrière, Paris, France; d Department of Neurology, Paris ALS Center, Pitie-Salpetriere University Hospital - Assistance Publique - Hopitaux de Paris, France; e Service de Pneumologie (Département R3S), AP-HP, Groupe Hospitalier Universitaire APHP-Sorbonne Université, Hôpital Pitié- Salpêtrière, Paris, F-75013, France; f University Paris Cité, Inserm UMR_S1124, 75270, Paris Cedex 06, France; g Sorbonne Université, INSERM, UMRS1158 Neurophysiologie Respiratoire Expérimentale et Clinique, Paris, France; h AP-HP, Groupe Hospitalier Universitaire APHP-Sorbonne Université, site Pitié-Salpêtrière, Service de Médecine Intensive et Réanimation (Département R3S), F-75013 Paris, France; i INSERM, UMRS1158 Neurophysiologie respiratoire expérimentale et clinique, Paris, France; j Groupe de Recherche Clinique en REanimation et Soins intensifs du Patient en Insuffisance Respiratoire aiguE (GRC-RESPIRE), Sorbonne Université, Paris, France; k Brain Liver Pitié-Salpêtrière Study Group, INSERM UMR_S 938, Centre de recherche Saint-Antoine, Maladies métaboliques, biliaires et fibro-inflammatoire du foie, Institute of Cardiometabolism and Nutrition, Paris, France

**Keywords:** Amyotrophic lateral sclerosis, Acute respiratory failure, Intensive care unit

## Abstract

**Background:**

Data on patients with amyotrophic lateral sclerosis (ALS) admitted to the intensive care unit (ICU) for acute respiratory failure are scarce, and early prognostic markers are lacking.

**Methods:**

We conducted a retrospective cohort study of consecutive patients with ALS admitted for acute respiratory failure to two ICUs of a tertiary referral center between 2016 and 2022. The primary outcome was 3-month mortality. Factors associated with 3-month mortality were assessed using multivariable Cox proportional hazards regression. Survival and functional outcomes were evaluated up to 12 months.

**Results:**

Among 139 patients [median age 66 (IQR 57–73) years, 61.2% being men], 109 (78.4%) patients presented with hypercapnia and 82 (59.0%) with hypoxemia (defined as PaO₂/FiO₂ ratio < 300) at ICU admission, with frequent overlap between both conditions. Non-invasive ventilation was used in 79.9% of patients, while 13.7% were intubated, and 2.2% underwent tracheostomy. Three-month mortality was 48.2%, increasing to 61.2% and 74.1% at 6 and 12 months respectively. In multivariable analysis, hypoxemia at ICU admission was independently associated with increased 3-month mortality (HR = 2.27, 95% CI: 1.28–4.01; *P* = 0.005), along with older age (HR=1.03 per year, 95% CI: 1.00–1.06; *P* = 0.035). Among patients alive at 3 months, a majority (79.2%) were living at home.

**Conclusion:**

ICU admission for acute respiratory failure in ALS is associated with substantial short-term mortality, consistent with the poor prognosis inherent to the natural history of the disease. Hypoxemia at admission identifies a subgroup at particularly high risk of death and may help inform early prognostic assessment and shared decision-making. However, patients who survive the acute ICU phase frequently achieve favorable short-term outcomes, with most living at home at 3 months.

## Introduction

Amyotrophic lateral sclerosis (ALS) is a progressive neurodegenerative disease characterized by motor neuron degeneration, leading to progressive muscle weakness, respiratory failure, and death [[1], [2], [3]]. Median survival from symptom onset ranges from 2 to 4 years, and respiratory failure represents the leading cause of death [1,3]; in this context, non-invasive ventilation (NIV) has become a cornerstone of care, as it improves survival and quality of life in patients with chronic respiratory insufficiency [4]. However, when patients present with acute respiratory failure (ARF), the optimal management strategy remains uncertain, particularly regarding the use of invasive mechanical ventilation and tracheostomy.

The literature on patients with ALS (pwALS) admitted to the intensive care unit (ICU) for ARF is limited. Only a few retrospective studies have reported outcomes in this population, suggesting high short-term mortality and substantial long-term disability among survivors [5,6]. However, data on medium- and long-term evolution after ICU discharge, including survival, functional status, and the need for prolonged ventilatory support, are scarce. Identifying prognostic factors in pwALS requiring ICU admission for ARF is crucial to inform decision-making, guide discussions with patients and families, and improve individualized care plans.

In this context, we conducted a retrospective study of pwALS admitted to two ICUs for ARF. The primary objective was to measure 3-month survival among pwALS admitted to the ICU for acute respiratory failure. Secondary objectives included assessing the use of invasive mechanical ventilation and tracheostomy, as well as survival and functional outcomes at 3, 6 and 12 months.

## Patients and methods

### Study design and patient selection

This retrospective observational study was conducted in two ICUs of the Pitié-Salpêtrière Hospital, which houses Paris ALS Center, one of the largest French referral ALS centers. All consecutive patients diagnosed with ALS who were admitted between January 1, 2016, and December 31, 2022 to the ICU for ARF were included, including patients in whom ALS was diagnosed during the ICU stay. Patients were identified from a comprehensive hospital database using the ICD-10 code for ALS (G122). Diagnosis of ALS was established by expert neurologists according to the revised El Escorial criteria [7]. ARF was defined as respiratory failure associated with hypercapnia (PaCO₂ > 45 mmHg), hypoxemia (ratio of partial pressure of oxygen to fraction of inspired oxygen (PaO₂/FiO₂) < 300) or both. For patients receiving supplemental oxygen via nasal cannula or face mask, FiO₂ was estimated using a standard approximation for nasal oxygen delivery (FiO₂ ≈ 21% + 4 × oxygen flow rate [L/min]).

Patients could initially have been admitted to another ICU before being transferred to our center. In cases of multiple ICU admissions, only the first admission was considered for analysis. Patients with a duration since ALS disease onset longer than 20 years were excluded, to limit heterogeneity related to atypical slow disease progression. Patients with no arterial gas at ICU admission and patients with a pre-existing tracheostomy were also excluded.

Patients were subsequently dichotomized into two groups based on their admission oxygenation status: group with hypoxemia defined by PaO₂/FiO₂ ratio < 300 (regardless of PaCO₂ levels), and group without hypoxemia defined by PaO₂/FiO₂ ratio ≥ 300.

The study was approved by the ethics committee of French Intensive Care Society (CE SRLF 23-064), all procedures followed the ethical standards of the institutional and national research committees and the principles of the Declaration of Helsinki (1975). It was reported in accordance with the Strengthening the Reporting of Observational Studies in Epidemiology (STROBE) guidelines, and the corresponding checklist is provided in Table S1.

### Data collection

In each ICU, data were extracted from electronic medical records. At the time of ICU admission, the following variables were recorded: date of ALS diagnosis, disease duration from onset, history of bulbar symptoms, Modified Rankin Scale (mRS) score, prior home non-invasive ventilation (NIV) prescription (which referred to patients for whom NIV had been prescribed by the neurology team, regardless of actual daily use), daily NIV use in hours (none, <12 h/day, and ≥12 h/day), gastrostomy before admission, date of hospital and ICU admission, date of discharge from ICU, etiology of ARF, Glasgow coma scale, temperature, arterial blood gas (PaO_2_, PaCO_2_, lactate, HCO3) with presence or absence of hypercapnia and hypoxemia, chloride, Simplified Acute Physiologic Score II (SAPS II) [8], Sepsis-related Organ Failure Assessment (SOFA) [9]. The use of NIV, invasive mechanical ventilation, tracheostomy or gastrostomy during the stay in ICU was collected. Finally, the following outcomes were assessed: ICU and in-hospital mortality after ICU admission, discharge destination, survival status and mRS scores at 3-, 6-, and 12-month after ICU admission. For patients initially admitted to another ICU and subsequently transferred to our center, the ICU admission date was defined as the date of admission to the first ICU.

### Outcomes

The primary outcome was 3-month mortality following ICU admission. Secondary outcomes included ICU mortality and in-hospital mortality, as well as mortality at 6 and 12 months after ICU admission. Causes of death occurring during ICU stay were also documented. Additional secondary outcomes were the use of invasive mechanical ventilation, duration of invasive ventilation, performance of tracheostomy or gastrostomy, ICU length of stay, occurrence of septic shock during ICU stay. Long-term disability was assessed using the Modified Rankin Scale (mRS), and living status (home, institutional care, or death) was recorded at 3, 6, and 12 months. Data were collected up to 12 months after ICU admission.

### Statistical analysis

Results were expressed as median (interquartile range) for continuous variables and number (percentage) for categorical variables. Comparisons between groups were performed using the Wilcoxon test for continuous variables and the Chi-square test for categorical variables.

Factors independently associated with 3-month mortality were assessed using a multivariable Cox proportional hazards regression model. Covariates were selected a priori based on clinical relevance and the number of events. The primary model included age at baseline, duration since disease onset, sex, baseline body mass index (BMI), history of bulbar symptoms, the presence of hypoxemia at ICU admission and prior home NIV prescription. Sensitivity analyses were performed by replacing prior home NIV prescription with daily NIV use (no use, <12 h/day, and ≥12 h/day), and by replacing duration since disease onset with ALS diagnosis during the ICU stay. Hazard ratios (HRs) are reported with 95% confidence intervals (CI). To visually explore the effects of significant factors, Kaplan-Meier survival curves for mortality from ICU admission were generated and compared using the log-rank test.

Long-term disability and living status were assessed at 3, 6, and 12 months using the mRS and place of residence (home vs institutional care). Trajectories over time were explored visually using a Sankey (alluvial) diagram showing transitions between mRS categories and living situation across follow-up time points.

All statistical analyses were performed using R software version 4.0.3 (R Core Team 2020; https://www.R-project.org/). Missing data were not imputed, and analyses were conducted using complete-case data. A two-sided p-value < 0.05 was considered statistically significant.

## Results

Fig. 1 shows that over the 7-year study period, 209 pwALS were admitted to one of the two participating ICUs, including 146 for ARF. Among them, 1 was excluded for duration since disease onset, 2 for missing arterial blood gas, and 4 because of pre-existing tracheostomy. Ultimately, 139 pwALS admitted to ICU for ARF were included in the final analysis.

**Fig. 1:**
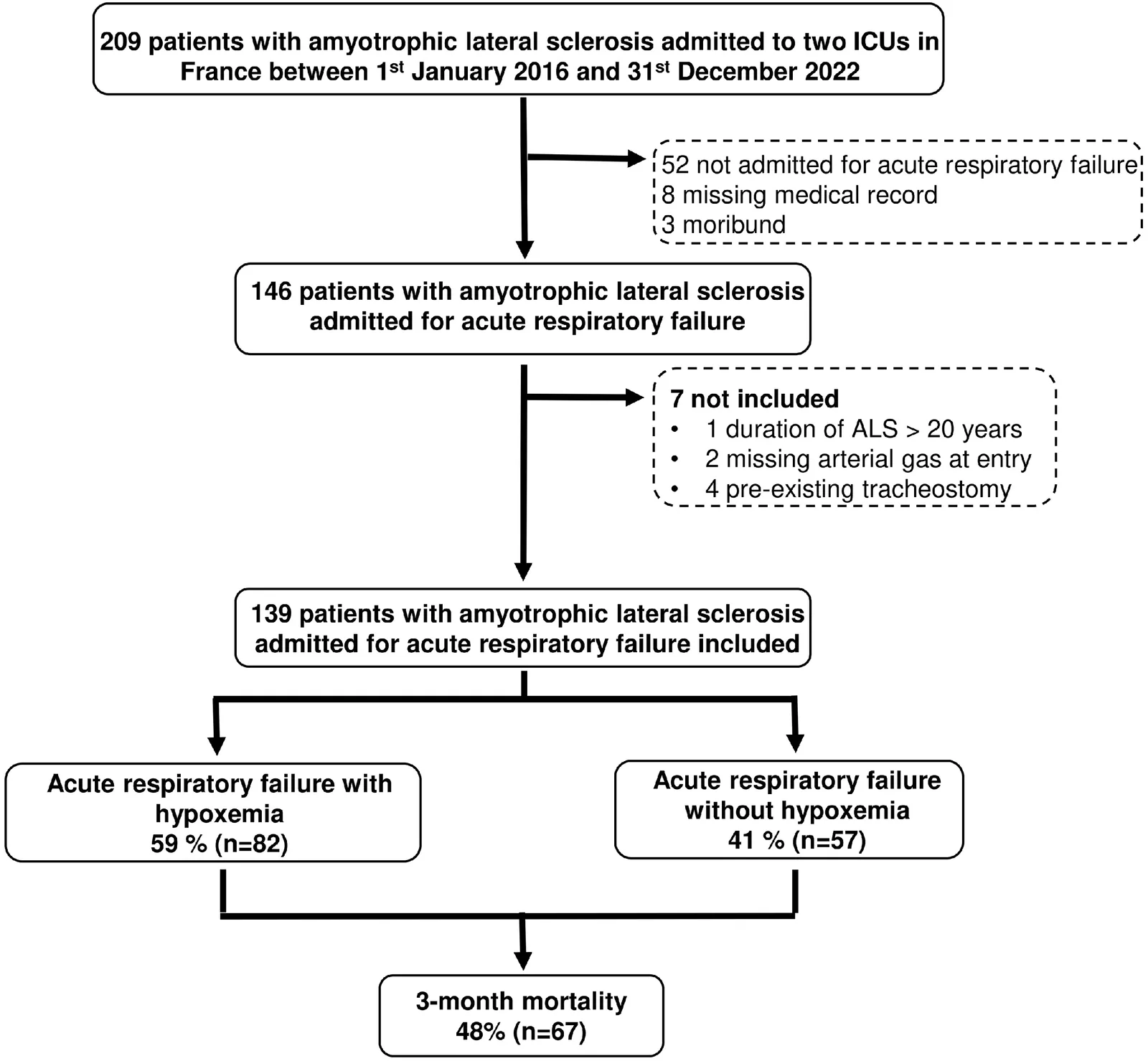
Flow chart of the study. ICU, intensive care unit; ALS, amyotrophic lateral sclerosis.

### Patient characteristics

Patients’ characteristics are presented in Table 1. The median age at ICU admission was 66 (57–73) years, with 61.2% being men. The median duration between disease onset and ICU admission was 25 (IQR : 14–43) months. Six (4.3%) patients had genetic ALS, and 96 (69.1%) had a history of bulbar symptoms. The median modified Rankin score was 3 (2–4). Before admission, 51 (36.7%) patients had prior home NIV prescription. Among them, 42 were actually using NIV, whereas 9 were not. Among patients with available daily NIV use data (n = 136), 14 (10.3%) used NIV for <12 h/day and 28 (20.6%) for ≥12 h/day. Twenty-six (18.7%) patients had gastrostomy placement. Twenty patients (14.4%) were newly diagnosed with ALS during their ICU stay.

**Table 1:** Patients’ characteristics at admission to the intensive care unit.

Characteristics	All patients (n = 139)	3–month survivors (n = 72)	3–month non–survivors (n = 67)	*P-*value
Age at ICU admission (years)	66 (57–73)	65 (55–73)	68 (60–73)	0.069
Male sex	85 (61)	40 (56)	45 (67)	0.219
ALS characteristics				
Bulbar symptoms	96 (69)	48 (67)	48 (72)	0.652
Genetic ALS form	6 (4)	5 (7)	1 (1)	0.245
Duration since disease onset (months)	25 (14–43)	27 (12–43)	24 (17–38)	0.784
ALS diagnosis in ICU	20 (14)	14 (19)	6 (9)	0.129
Prior home NIV prescription	51 (37)	26 (36)	25 (37)	>0.999
Daily NIV use^a^				0.287
None	94 (69)	48 (68)	46 (71)	
Under 12 h per day	14 (10)	10 (14)	4 (6)	
Above 12 h per day	28 (21)	13 (18)	15 (23)	
Gastrostomy before ICU admission	26 (19)	12 (17)	14 (21)	0.674
Rankin score at ICU admission	3 (2–4)	3 (2–4)	3 (2–4)	0.231

Data are expressed as median (interquartile range) for quantitative variables and as *n*(%) for qualitative variables.

ARF, acute respiratory failure; ALS, amyotrophic lateral sclerosis; NIV, non-invasive ventilation ; ICU, intensive care unit.

a Daily NIV use data were available for 136 patients (71 3-month survivors and 65 3-month non-survivors). Percentages were calculated using the number of patients with available data in each group as the denominator. ‘None’ includes 9 patients with a prior home NIV prescription who were not using NIV.

### ICU admission and management

ICU admission characteristics and management are detailed in Table 2. The median BMI was 20 (18–24). At ICU admission, 109 (78.4%) patients had hypercapnia on arterial gas and 82 (59.0%) had hypoxemia. Arterial blood gas revealed a median PaCO_2_ of 52 (46–67) mmHg, a PaO_2_/FiO_2_ ratio of 276 (169–372) and a lactate level of 1.1 (0.8–1.4) mmol/L. The median SAPS II score was 34 (27–41), and the median SOFA score was 1 (0–3). Compared with patients without hypoxemia, those with hypoxemia at ICU admission were more likely to be men and had lower PaCO₂, lower HCO3-, lower chloride and higher lactate levels at ICU admission (Table S2 and S3 in the Online supplement).

**Table 2:** Intensive care unit admission and management, comparison between 3–month survivors and non–survivors.

Characteristics	All patients (n = 139)	3–month survivors (n = 72)	3–month non–survivors (n = 67)	*P*-value
Respiratory abnormalities at ICU admission^a^				
Hypercapnia	109 (78)	59 (82)	50 (75)	0.495
Hypoxemia^b^	82 (59)	33 (46)	49 (73)	**0.002**
ICU admission data				
BMI	20.4 (18–24)	20.6 (18–25)	20.2 (18–24)	0.624
Glasgow coma scale	15 (15–15)	15 (15–15)	15 (14–15)	**0.036**
Temperature (°C)	36.6 (36.3–37.0)	36.8 (36.4–37.1)	36.5 (36.1–37.0)	0.086
pH	7.38 (7.31−7.43)	7.38 (7.32−7.44)	7.38 (7.31−7.42)	0.466
PaO_2_/FiO_2_	276 (169–372)	316 (240–386)	213 (146–323)	**<0.001**
PaCO_2_ (mmHg)	52 (46–67)	53 (47–71)	50 (46–62)	0.254
Lactate (mmol/L)	1.1 (0.8–1.4)	0.9 (0.7–1.3)	1.1 (0.9–1.6)	**0.033**
HCO3- (mmol/L)	30 (27–34)	31 (28–36)	29 (27–32)	**0.015**
Chloride (mmol/L)	100 (96–102)	100 (96–102)	100 (97–102)	0.487
SAPS II	34 (27–41)	31 (28–38)	37 (25–42)	0.124
SOFA	1 (0–3)	1 (0–2)	2 (0–3)	0.224
Respiratory management in the ICU				
Non–invasive ventilation	111 (80)	53 (74)	58 (87)	0.091
Intubation during ICU stay	19 (14)	9 (13)	10 (15)	0.866
Duration of invasive ventilation (days)	10 (5–18)	14 (10–19)	5 (3–14)	0.078
Tracheostomy during ICU stay	3 (2)	3 (4)	0 (0)	0.269
Septic shock during ICU stay	4 (3)	1 (1)	3 (4)	0.561
Gastrostomy during ICU stay	9 (6)	4 (6)	5 (7)	0.911
NIV at ICU discharge^c^	73 (69)	47 (65)	26 (76)	0.349
ICU length of stay (days)	3 (1–7)	4 (1–8)	3 (2−6)	0.567
In–ICU mortality^d^	33 (24)	–	–	–
In–hospital mortality after ICU discharge	18 (13)	–	–	–

Data are expressed as median (interquartile range) for quantitative variables and as *n*(%) for qualitative variables.

Bold values indicate statistical significance (P < 0.05).

ARF, acute respiratory failure; BMI, body mass index; PaO_2_/FiO_2_, partial pressure of arterial oxygen/fraction of inspired oxygen ratio, PaCO_2_, partial pressure of arterial carbon dioxide, HCO3–, bicarbonate; SAPS II, simplified acute physiology score; SOFA, sepsis–related organ failure assessment; ICU, intensive care unit.

a Patients can be admitted with hypercapnia and with hypoxemia. Hypercapnia is defined as PaCO_2_ > 45 mmHg, hypoxemia is defined as PaO_2_/FiO_2_ < 300.

b ARF etiologies were: with hypoxemia (n = 82), bacterial pneumonia (n = 47), undetermined (n = 28), COVID-19 pneumonia (n = 2), atelectasis (n = 2), and other (n = 3); without hypoxemia (n = 57), undetermined (n = 36), bacterial pneumonia (n = 8), aspiration (n = 5), bowel obstruction (n = 3), viral infection (n = 2), and other (n = 3).

c Out of 106 patients discharged from the ICU, 72 survivors at 3 months and 34 non-survivors.

d Among ICU deaths (n = 33), withdrawal of life-sustaining therapies preceded death in 30/33 (90.9%) patients, while 3/33 (9.1%) patients died from cardiac arrest.

NIV was used in the ICU in 111 (79.9%) patients, for a median of 4 (1–6) days (Table 2). During their ICU stay, 19 (13.7%) patients were intubated, and 3 (2.2%) patients underwent tracheostomy. A total of 9 (6.5%) patients underwent gastrostomy, and 4 (2.9%) developed septic shock during their stay. Median ICU length of stay was 3 (1–7) days.

ICU mortality was 23.7%, and in-hospital mortality after ICU discharge was 12.9%. Among ICU deaths, withdrawal of life-sustaining therapies preceded death in 30/33 (90.9%) patients, while unexpected cardiac arrest accounted for 3/33 (9.1%) deaths. Among patients who died in the ICU, withdrawal of life-sustaining therapies preceded death in 23/26 (88.5%) patients with hypoxemia and in all 7 patients without hypoxemia (Fisher’s exact test, P = 1.00).

### Patients requiring invasive mechanical ventilation

Characteristics of patients requiring invasive mechanical ventilation are summarized in Table 3. Among the 19 patients, 9 (47.4%) were intubated prior to ICU transfer. Indications for intubation were hypercapnia (57.9%), hypoxemia (31.6%), and cardio-respiratory arrest (10.5%). Notably, although hypercapnia was the most frequently reported indication for intubation, the majority of intubated patients (84.2%) also presented with hypoxemia at ICU admission (Table S4). Median duration of invasive mechanical ventilation was 10 days (5–18), and median ICU length of stay was 19 (8–35) days. Among the 19 patients receiving invasive mechanical ventilation, 3 (15.8%) underwent tracheostomy and 7 (36.8%) died in the ICU.

**Table 3:** Characteristics of patients requiring invasive mechanical ventilation, comparison between 3–month survivors and non–survivors.

Characteristics	Intubated patients (n = 19)	3–month survivors (n = 9)	3–month non–survivors (n = 10)^a^	*P-*value
Age at ICU admission (years)	64 (57–69)	62 (55–69)	66 (60–69)	0.780
Male sex	14 (74)	7 (78)	7 (70)	>0.999
Bulbar symptoms	8 (42)	4 (44)	4 (40)	>0.999
Duration since disease onset (months)	17 (12–44)	17 (10–51)	20 (14–39)	0.842
ALS diagnosis in ICU	7 (37)	4 (44)	3 (30)	0.861
Intubation in another ICU	9 (47)	5 (56)	4 (40)	0.827
Delay between ICU admission and intubation (days)	0 (0–1)	0 (0–1)	0 (0–2)	0.723
Indication for intubation				
Hypoxemia	6 (32)	3 (33)	3 (30)	>0.999
Hypercapnia	11 (58)	6 (67)	5 (50)	0.788
Arterial gas at intubation				
pH	7.36 (7.26−7.44)	7.36 (7.27−7.46)	7.35 (7.26−7.40)	0.806
PaO_2_ (mmHg)	80 (64–94)	77 (66–86)	81 (64–94)	0.743
PaCO_2_ (mmHg)	56 (41–72)	64 (43–95)	52 (39–66)	0.286
Lactate (mmol/L)	1.1 (0.7–1.3)	1.1 (0.6–1.2)	1.2 (0.8–1.3)	0.604
HCO3- (mmol/L)	29 (25–31)	29 (25–40)	27 (26–30)	0.530
Invasive ventilation duration (days)	10 (5–18)	14 (10–19)	5 (3–14)	0.078
Tracheostomy during ICU stay	3 (16)	3 (33)	0 (0)	0.174
ICU length of stay (days)	19 (8–35)	32 (13–40)	13 (4–26)	0.094
NIV at discharge^b^	8/12 (67)	5/9 (56)	3/3 (100)	0.480
In-ICU mortality	7 (37)	–	–	–
In-hospital mortality after ICU discharge	2 (11)	–	–	–

Data are expressed as median (interquartile range) for quantitative variables and as *n*(%) for qualitative variables.

ICU, intensive care unit; PaO_2_, partial pressure of arterial oxygen ; PaCO_2_, partial pressure of arterial carbon dioxide.

a Among 3-month non-survivors (n = 10): 7 died in the ICU, 2 in hospital, and 1 after discharge.

b Out of 12 intubated patients discharged from the ICU, 9 being alive at 3 months.

### Outcomes at 3, 6, and 12 months

Overall 3-month mortality was 48.2%. Mortality was 61.2% at 6 months and 74.1% at 12 months. At all time points, mortality was significantly higher among patients admitted for ARF with hypoxemia than among those admitted without hypoxemia (Table S5).

Table 1, Table 2, Table 3 outline identified factors associated with 3-month mortality. In the multivariate analysis, two factors were independently associated with 3-month mortality: older age at ICU admission (hazard ratio [HR] = 1.03, 95% CI: 1.00–1.06; *P* = 0.035) and the presence of hypoxemia at ICU admission (HR=2.27, 95% CI: 1.28–4.01; *P* = 0.005) (Table 4). Sex, duration since disease onset, BMI, history of bulbar symptoms, and prior home NIV prescription were not independently associated with mortality. In a sensitivity analysis substituting prior home NIV prescription by the daily NIV use (no use, <12 h/day, and ≥12 h/day) (Table S6), age and hypoxemia at ICU admission remained independently associated with 3-month mortality, while daily NIV use was not. Similar results were observed when duration since disease onset was replaced by ALS diagnosis during the ICU stay, which was not independently associated with 3-month mortality (Table S7).

**Table 4:** Factors associated with 3–month mortality, multivariate survival analysis.

Variable	HR (95% CI)	*P-*value
Age, *per year increase*	1.03 (1.00−1.06)	**0.035**
Sex, *male*	1.06 (0.62−1.80)	0.843
Duration since disease onset, *per year increase*	1.04 (0.94−1.15)	0.406
Bulbar symptoms	1.07 (0.60−1.90)	0.812
BMI, *per point increase*	0.97 (0.91−1.03)	0.303
Hypoxemia at ICU admission	2.27 (1.28−4.01)	**0.005**
Prior home NIV prescription	1.08 (0.63−1.87)	0.780

Hazard ratio (HR) and 95% confidence intervals (CI) computed using multivariate survival analysis.

Bold values indicate statistical significance (P < 0.05).

BMI, body mass index ; NIV, non-invasive ventilation.

Fig. 2 illustrates cumulative survival probability up to 3 months, stratified by presence of hypoxemia at ICU admission. Fig. 3 illustrates trajectory over time, regarding mRS and living status. At 3 months, 57 of the 72 survivors (79.2%) were living at home; living status was unknown for 15 survivors (20.8%).

**Fig. 2:**
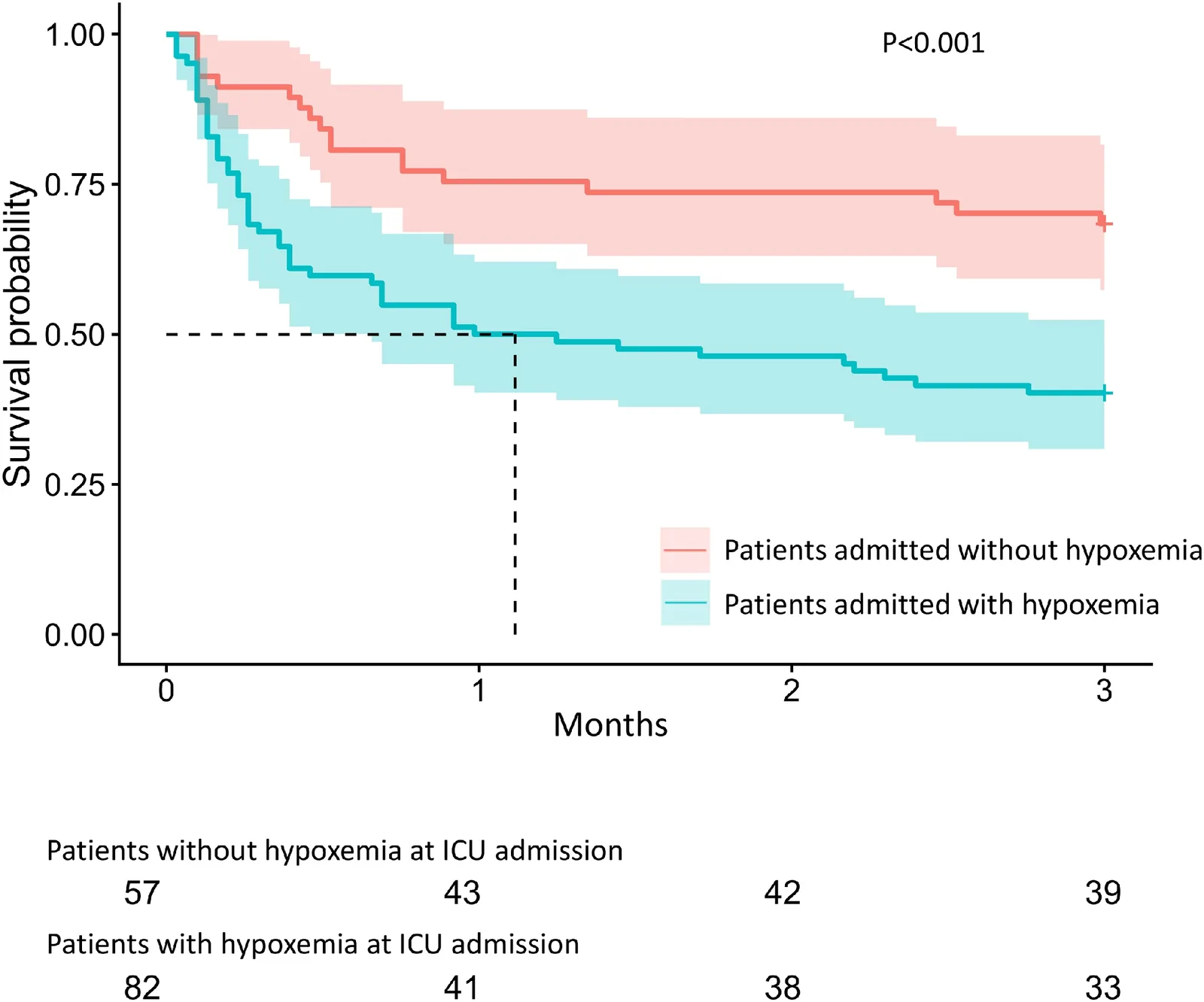
Cumulative survival up to 3 months in patients with and without hypoxemia at ICU admission. ICU, intensive care unit.

**Fig. 3:**
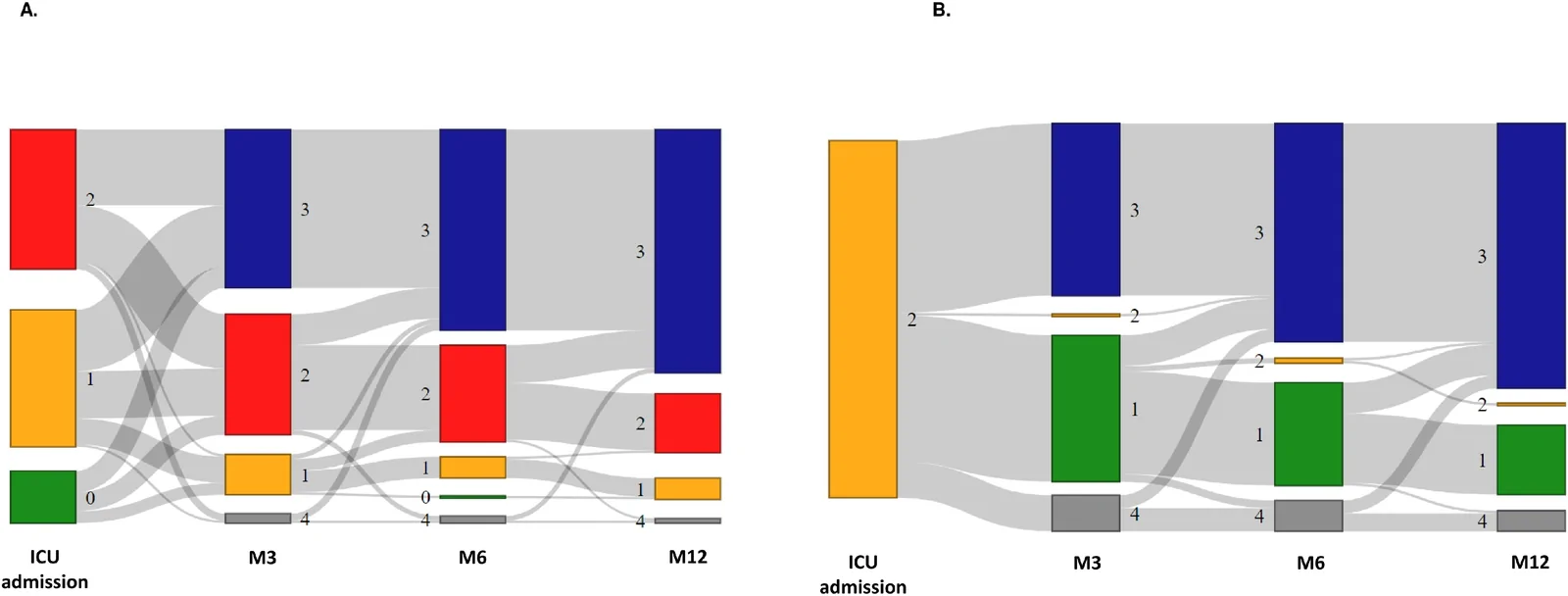
Trajectory over time using an alluvial diagram of A) modified Rankin Scale score; B) living situation. A: green: Rankin 0–1 ; yellow: Rankin 2–3 ; red: Rankin 4–5 ; blue: Death. B: green: Home; yellow: Hospital/rehabilitation facility; blue: Death; grey: Unknown. ICU, intensive care unit.

## Discussion

The main findings of our study can be summarized as follows: (1) mortality at 3 months after ICU admission for ARF in pwALS approached 50%, a figure that should be interpreted in light of the natural history of ALS; (2) hypoxemia at ICU admission is frequent and strongly associated with short- and mid-term mortality; (3) despite the severity of illness, more than half of patients (51.8%) were alive at 3 months after ICU admission; and (4) among patients alive at 3 months, the majority were living at home, including those who had required invasive mechanical ventilation.

The characteristics of our cohort are consistent with those reported in previous studies of pwALS admitted to the ICU, namely a predominance of older male patients with relatively short disease duration and significant baseline disability [6,10]. Non-invasive ventilation was used in the majority of patients in the ICU, particularly in cases of hypercapnic respiratory failure related to respiratory muscle weakness and restrictive ventilatory impairment, consistent with its established role in the management of chronic respiratory insufficiency in ALS [4,[11], [12], [13], [14]]. The proportion of genetic ALS in our cohort (4.3%) was lower than the ∼10–15% typically reported in ALS populations [2], which may be explained by the fact that some patients may not yet have undergone comprehensive genetic testing, potentially leading to an underestimation of genetic cases.

Interestingly, only 36.7% of patients had prior home NIV prescription before ICU admission. While this proportion is consistent with contemporary ALS cohorts reported in the literature [15], one might have expected a substantially higher prevalence of NIV use in a population admitted to the ICU for acute respiratory failure. This may reflect relatively rapid respiratory deterioration in a substantial proportion of the cohort, as illustrated by the fact that 14.4% of patients were newly diagnosed with ALS during their ICU stay and therefore had no prior indication for NIV.

Three-month mortality in our cohort reached 48.2%, which is in agreement with the mortality rates previously reported in pwALS admitted to the ICU for respiratory failure [6]. Although substantial, this mortality must be interpreted in the context of the natural history of ALS, a progressive disease with a median survival of approximately 2–3 years from symptom onset [1,2]. In addition, our cohort represented a relatively advanced ALS population, with a median disease duration of 25 months at ICU admission, which may partly explain the observed mortality. Hospitalization for respiratory failure has been identified as a major prognostic turning point in ALS, being associated with a sharp increase in subsequent mortality [5]. Beyond mortality, ICU admission for acute respiratory failure in ALS raises major ethical and prognostic issues, given the risk of prolonged ventilatory dependence and severe functional impairment among survivors.

Although overall mortality was significant, our results highlight the marked heterogeneity of outcomes among pwALS admitted to the ICU for acute respiratory failure. Hypoxemia at ICU admission identified a subgroup of patients with particularly poor prognosis, whereas patients without hypoxemia had significantly lower mortality at all follow-up time points. To our knowledge, this is the first study to identify hypoxemia as a prognostic factor in this population. Notably, this association persisted despite the use of a PaO₂/FiO₂ threshold of <300 to define hypoxemia, thereby including patients with only mild oxygenation impairment. These findings suggest that hypoxemia reflects a distinct and more severe pathophysiological profile, likely related to superimposed pulmonary complications such as aspiration pneumonia, atelectasis, or impaired airway clearance, rather than isolated ventilatory pump failure. Treatment-limitation decisions may have influenced the association between hypoxemia and mortality, as greater respiratory severity could potentially prompt earlier withdrawal of life-sustaining therapies. This concern is further supported by the shorter duration of invasive mechanical ventilation among non-survivors, which may partly reflect earlier treatment limitation. However, withdrawal of life-sustaining therapies was similarly frequent among ICU deaths in patients with and without hypoxemia. Although this finding argues against treatment limitation as the sole explanation for the association between hypoxemia and mortality, residual confounding cannot be excluded given the retrospective design and the small number of ICU deaths.

In the previous cohort by Mayaux et al. [6], severity of hypoventilation at ICU admission, reflected by higher PaCO₂ and lower pH, was associated with 3-month mortality. In contrast, hypercapnia was not associated with outcome in our cohort, whereas hypoxemia, which was not specifically evaluated as a prognostic factor in the previous study, emerged as the main respiratory prognostic marker.

Importantly, our study also shows that ICU admission is not uniformly futile in this population. Among patients alive at 3 months, a majority (79.2%) were living at home, although living status was unknown for 20.8%. In addition, the median ICU length of stay was short (3 days), suggesting that, in the absence of early death, ICU management may consist of a relatively brief and limited intervention allowing rapid return to the home environment. This finding is clinically meaningful, as returning home likely reflects social reintegration, and the possibility for patients to continue their disease trajectory in their usual environment. However, longitudinal mRS data suggested substantial disability among survivors, although limited follow-up data precluded firm conclusions regarding functional deterioration over time. These results suggest that, in selected pwALS, ICU management for acute respiratory failure may allow meaningful survival rather than merely prolonging the dying process. Among patients requiring invasive mechanical ventilation, ICU mortality remained high, reflecting the severity of respiratory failure in this subgroup. Notably, all ICU deaths among intubated patients occurred in those presenting with hypoxemia at ICU admission, consistent with the association between hypoxemia and poorer outcomes observed in the overall cohort.

Age was the second factor independently associated with mortality, consistent with previous studies identifying older age as a robust prognostic factor in ALS [[16], [17], [18], [19]]. In contrast, baseline ALS-related characteristics such as duration since disease onset, functional status, bulbar symptoms or prior home NIV prescription were not independently associated with mortality. Notably, the daily use of NIV at home was not associated with outcome, challenging the common assumption that more advanced chronic ventilatory dependence necessarily portends poorer short-term prognosis after ICU admission.

Taken together, these findings emphasize the need for individualized decision-making in pwALS admitted to the ICU for acute respiratory failure. While hypoxemic presentations are associated with a particularly poor prognosis and may warrant cautious discussions regarding treatment intensity, other patients, especially those with isolated hypercapnia, may benefit from ICU support, with a realistic possibility of returning home and maintaining meaningful life trajectories. Hypoxemia at ICU admission may therefore represent a simple and clinically relevant marker to guide prognostic assessment and shared decision-making with patients and families. Importantly, functional impairment should not be equated with poor quality of life in ALS. Because of the disability paradox, patients with advanced disability may report a better quality of life than expected from their degree of physical impairment [20]. This discrepancy is particularly relevant in the acute ICU setting, where patient-perceived quality of life may be difficult to assess. Therefore, functional scales such as the mRS should not be used alone to guide treatment-limitation decisions, which should also take into account patients’ values, preferences, and previously expressed wishes [21]. Decisions regarding treatment intensity should also consider their implications for patients’ families. Caregiver burden and quality of life are important considerations in ALS [22], particularly when prolonged ventilatory support is contemplated, as tracheostomy ventilation may profoundly affect caregivers’ autonomy, family life, and home-care responsibilities [23]. These considerations should be integrated into shared decision-making with patients and their relatives.

Our study has several strengths. It represents one of the largest cohorts of pwALS admitted to the ICU for acute respiratory failure, with detailed characterization of respiratory failure phenotypes and medium-term follow-up. The systematic assessment of hypoxemia and hypercapnia at ICU admission allowed us to identify clinically relevant prognostic profiles. However, several limitations must be acknowledged. First, the retrospective design may have introduced selection and information bias. In particular, data on pwALS evaluated for but not admitted to the ICU were unavailable, precluding assessment of potential selection bias related to ICU triage or treatment-limitation decisions. Second, the study was conducted in a tertiary referral center with extensive experience in ALS care, which may limit generalizability. Third, detailed ventilatory parameters were not available. Fourth, detailed information regarding the timing and reasons for decisions to limit life-sustaining therapies, as well as patients’ previously expressed wishes, was not systematically available, precluding a comprehensive assessment of their potential influence on mortality. Finally, functional outcomes beyond the modified Rankin Scale and quality-of-life measures were not systematically assessed, as disease-specific functional scores such as the ALSFRS-R were available for only a limited subset of patients.

## Conclusion

Acute respiratory failure in pwALS admitted to the ICU is associated with significant short-term mortality, reflecting the poor prognosis inherent to the natural history of ALS. Hypoxemia at ICU admission is a major prognostic factor, identifying patients at particularly high risk of death. However, patients who survive the acute ICU phase frequently return home after a relatively short ICU stay. These findings may help refine prognostic assessment and support individualized decisions regarding ICU admission and treatment intensity.

## CRediT authorship contribution statement

A.C.: Conceptualization, Data curation, Investigation, Formal analysis, Writing – original draft.

R.B.: Data curation, Investigation.

A.L.: Formal analysis, Visualization, Writing – review & editing.

S.H.: Data curation, Investigation, Writing – review & editing.

A.H. and C.M.: Conceptualization, Investigation, Writing – review & editing.

G.B., C.M.P., M.P., M.P., L.L.G., N.W., S.D. and J.M.: Investigation, Writing – review & editing.

All authors reviewed the manuscript, critically revised it for important intellectual content, approved the final version, and agree to be accountable for all aspects of the work.

## Ethics approval and consent to participate

The study was approved by the ethics committee of French Intensive Care Society (CE SRLF 23−064), all procedures followed the ethical standards of the institutional and national research committees and the principles of the Declaration of Helsinki (1975).

## Funding

None.

## Data availability

The datasets used and/or analysed during the current study are available from the corresponding author on reasonable request.

## Declaration of competing interests

M.P reports congress registration fees and speaker fees from Air Liquide Medical Systems, outside the submitted work.

N.W reports consultant fees from Alexion and Lectures fees from Lucane Pharma and Sedana, outside the submitted work.

C.MP has nothing to disclose regarding the present study. Outside this study, she reports over the last 3 years research grant from Chiesi France, personal fees for consulting and teaching activities from Chiesi France, Sanofi France, Menarini France, GSK France, Oxylis, SOS Oxygène, ResMed France, Fisher and Paykel, Breas, and Lowenstein Medical France, support for attending meeting from ASV, Asten, SOS Oxygène.

No other author report any competing interests.
