# Supplementary material for: Outcomes of patients with amyotrophic lateral sclerosis admitted to the ICU for acute respiratory failure
Source: Ann Intensive Care. 2026 Oct 12;16:100183. doi: 10.1016/j.aicoj.2026.100183 (PMC13695101; doi:10.1016/j.aicoj.2026.100183)
Supplement: Supplementary file 1 [file mmc1.docx]

**Characteristics and prognosis of amyotrophic lateral sclerosis patients admitted to the ICU for acute respiratory failure**

**Online supplement**

**Figure S1. Trajectory over time for patient surviving after intubation using an alluvial diagram of A) modified Rankin Scale score; B) living situation**

**Table S1. STROBE Statement—Checklist of items included in our study**

**Table S2. Patients characteristics at admission to the intensive care unit, comparison between patients with and without hypoxemia**

**Table S3. Intensive care unit management, comparison between patients with and without hypoxemia**

**Table S4. Characteristics of intubated patients, comparison between patients with and without hypoxemia**

**Table S5. Follow-up outcome after ICU admission, comparison between patients with and without hypoxemia**

**Table S6. Sensitivity analysis, factors associated with 3-month mortality, multivariate survival analysis**

**Figure S1. Trajectory over time for the 10 patients surviving after invasive mechanical ventilation and without tracheostomy, using an alluvial diagram of A) modified Rankin Scale score; B) living situation**

A: green: Rankin 0–1 ; yellow: Rankin 2–3 ; red: Rankin 4–5 ; blue: Death

B: green: Home; yellow: Hospital/rehabilitation facility; blue: Death; grey: Unknown

ICU, intensive care unit

**A.**

**ICU admission**

**ICU admission**

**M3**

**M12**

**M6**

**M3**

**M6**

**M12**

**B.**

**Table S1. STROBE Statement—Checklist of items included in our study.**

|  | Item No | Recommendation | Reported on page/section |
| --- | --- | --- | --- |
| **Title and abstract** | 1 | (*a*) Indicate the study’s design with a commonly used term in the title or the abstract | **Title** and **abstract** |
|  |  | (*b*) Provide in the abstract an informative and balanced summary of what was done and what was found | **Abstract** |
| Introduction | | |  |
| Background/rationale | 2 | Explain the scientific background and rationale for the investigation being reported | **Introduction** |
| Objectives | 3 | State specific objectives, including any prespecified hypotheses | **Introduction** |
| Methods | | |  |
| Study design | 4 | Present key elements of study design early in the paper | **Patients and Methods**  Study design and patients selection |
| Setting | 5 | Describe the setting, locations, and relevant dates, including periods of recruitment, exposure, follow-up, and data collection | **Patients and Methods**  Study design and patients selection |
| Participants | 6 | (*a*) Give the eligibility criteria, and the sources and methods of selection of participants. Describe methods of follow-up | **Patients and Methods**  Study design and patients selection |
|  |  | (*b*) For matched studies, give matching criteria and number of exposed and unexposed |  |
| Variables | 7 | Clearly define all outcomes, exposures, predictors, potential confounders, and effect modifiers. Give diagnostic criteria, if applicable | **Patients and Methods**  Outcomes |
| Data sources/ measurement | 8 | For each variable of interest, give sources of data and details of methods of assessment (measurement). Describe comparability of assessment methods if there is more than one group | **Patients and Methods**  Data collection |
| Bias | 9 | Describe any efforts to address potential sources of bias | **Patients and Methods**  Statistical analysis |
| Study size | 10 | Explain how the study size was arrived at | Non applicable |
| Quantitative variables | 11 | Explain how quantitative variables were handled in the analyses. If applicable, describe which groupings were chosen and why | **Patients and Methods**  Statistical analysis |
| Statistical methods | 12 | (*a*) Describe all statistical methods, including those used to control for confounding | **Patients and Methods**  Statistical analysis |
|  |  | (*b*) Describe any methods used to examine subgroups and interactions | **Patients and Methods**  Statistical analysis |
|  |  | (*c*) Explain how missing data were addressed | **Patients and Methods**  Statistical analysis |
|  |  | (*d*) If applicable, explain how loss to follow-up was addressed |  |
|  |  | (*e*) Describe any sensitivity analyses | **Patients and Methods**  Statistical analysis |
| Results | | |  |
| Participants | 13 | (a) Report numbers of individuals at each stage of study—eg numbers potentially eligible, examined for eligibility, confirmed eligible, included in the study, completing follow-up, and analysed | **Results**  **Fig 1** |
|  |  | (b) Give reasons for non-participation at each stage | **Results**  **Fig 1** |
|  |  | (c) Consider use of a flow diagram | **Fig 1** |
| Descriptive data | 14 | (a) Give characteristics of study participants (eg demographic, clinical, social) and information on exposures and potential confounders | **Results**  Patient’s characteristics |
|  |  | (b) Indicate number of participants with missing data for each variable of interest | **Tables** |
|  |  | (c) Summarise follow-up time (eg, average and total amount) | Non applicable |
| Outcome data | 15 | Report numbers of outcome events or summary measures over time | **Results** |
| Main results | 16 | (*a*) Give unadjusted estimates and, if applicable, confounder-adjusted estimates and their precision (eg, 95% confidence interval). Make clear which confounders were adjusted for and why they were included | **Results**  Follow-up |
|  |  | (*b*) Report category boundaries when continuous variables were categorized | Non applicable |
|  |  | (*c*) If relevant, consider translating estimates of relative risk into absolute risk for a meaningful time period | Non applicable |
| Other analyses | 17 | Report other analyses done—eg analyses of subgroups and interactions, and sensitivity analyses | **Results**  Follow-up |
| Discussion | | |  |
| Key results | 18 | Summarise key results with reference to study objectives | **Discussion** |
| Limitations | 19 | Discuss limitations of the study, taking into account sources of potential bias or imprecision. Discuss both direction and magnitude of any potential bias | **Discussion** |
| Interpretation | 20 | Give a cautious overall interpretation of results considering objectives, limitations, multiplicity of analyses, results from similar studies, and other relevant evidence | **Discussion** |
| Generalisability | 21 | Discuss the generalisability (external validity) of the study results | **Discussion** |
| Other information | | |  |
| Funding | 22 | Give the source of funding and the role of the funders for the present study and, if applicable, for the original study on which the present article is based | **Funding** |

**Table S2. Patients characteristics at admission to the intensive care unit for ARF, comparison between patients with and without hypoxemia**

| **Characteristics** | **ARF with hypoxemia**  **(n=82)** | **ARF without hypoxemia**  **(n=57)** | ***P*-value** |
| --- | --- | --- | --- |
| Age at ICU admission (years) | 66 (58–73) | 66 (56–73) | 0.717 |
| Male sex | 60 (73) | 25 (44) | **<0.001** |
| ALS characteristics |  |  |  |
| ALS spinal form | 49 (60) | 43 (75) | 0.082 |
| ALS bulbar form | 56 (68) | 40 (70) | 0.960 |
| Genetic ALS form | 2 (2) | 4 (7) | 0.378 |
| Duration since disease onset (months) | 24 (15–43) | 25 (13–40) | 0.709 |
| ALS diagnosis in ICU | 10 (12) | 10 (18) | 0.523 |
| Prior home NIV prescription | 30 (37) | 21 (37) | >0.999 |
| Daily NIV use^*^ |  |  | 0.737 |
| None | 57 (71) | 37 (66) |  |
| Under 12h per day | 7 (9) | 7 (13) |  |
| Above 12h per day | 16 (20) | 12 (21) |  |
| Gastrostomy before ICU admission | 15 (18) | 11 (19) | >0.999 |
| Rankin score at ICU admission | 3 (2–4) | 3 (2–4) | 0.084 |

Results are expressed as median (interquartile range) for quantitative variables and as number (percentage) for qualitative variables.

ARF, acute respiratory failure; ALS, amyotrophic lateral sclerosis; ICU, intensive care unit

^*^ Daily NIV use data were available for 136 patients (80 with hypoxemia and 56 without hypoxemia). Percentages were calculated using the number of patients with available data in each group as the denominator.

**Table S3. Intensive care unit admission and management,** **comparison between patients with and without hypoxemia**

|  | **ARF with hypoxemia**  **n = 82** | **ARF without hypoxemia**  **n = 57** | ***P*-value** |
| --- | --- | --- | --- |
| Hypercapnia at ICU admission | 52 (63) | 57 (100) | **<0.001** |
| ICU admission data |  |  |  |
| BMI | 20.8 (18–25) | 20.1 (17–24) | 0.242 |
| Glasgow coma scale | 15 (15–15) | 15 (15–15) | 0.191 |
| Temperature (°C) | 36.6 (36.2 – 37.0) | 36.8 (36.4 – 37.1) | 0.219 |
| pH | 7.38 (7.31-7.43) | 7.38 (7.32-7.42) | 0.885 |
| PaO_2_/FiO_2_ | 203 (136–244) | 386 (352-483) | **<0.001** |
| PaCO_2_ (mmHg) | 49 (42–68) | 56 (48–64) | **0.027** |
| Lactate (mmol/L) | 1.2 (0.8-1.6) | 0.9 (0.8-1.3) | **0.022** |
| HCO3– (mmol/L) | 29 (26–32) | 32 (28–35) | **0.011** |
| Chloride (mmol/L) | 100 (97–103) | 99 (95–101) | **0.037** |
| SAPS II | 34 (24–41) | 33 (28–39) | 0.952 |
| SOFA | 2 (0–3) | 1 (0–3) | 0.340 |
| Respiratory management |  |  |  |
| Non–invasive ventilation | 68 (83) | 43 (75) | 0.386 |
| Duration of non–invasive ventilation (days) | 3 (1–6) | 4 (2–7) | 0.116 |
| Intubation during ICU stay | 16 (20) | 3 (5) | **0.031** |
| Complications during ICU stay |  |  |  |
| Septic shock | 3 (4) | 1 (2) | 0.885 |
| Gastrostomy during ICU stay | 7 (9) | 2 (4) | 0.404 |
| NIV at ICU discharge^*^ | 39 (70) | 34 (68) | >0.999 |
| ICU length of stay | 3 (2–8) | 3 (1–6) | 0.488 |
| In–ICU mortality | 26 (32) | 7 (12) | **0.014** |
| In–hospital mortality after ICU discharge | 13 (16) | 5 (9) | 0.334 |

Results are expressed as median (interquartile range) for quantitative variables and as number (percentage) for qualitative variables.

ARF, acute respiratory failure; BMI, body mass index; PaO_2_/FiO_2_, partial pressure of arterial oxygen/fraction of inspired oxygen ratio, PaCO_2_, partial pressure of arterial carbon dioxide, HCO3-, bicarbonate; SAPS II, simplified acute physiology score; SOFA, sepsis-related organ failure assessment; ICU, intensive care unit

^*^ Out of 56 patients admitted with hypoxemia and discharged from the ICU, and 50 patients without hypoxemia and discharged from the ICU

**Table S4. Characteristics of patients requiring invasive mechanical ventilation, comparison between patients with and without hypoxemia**

|  | **ARF with hypoxemia**  **n = 16** | **ARF without hypoxemia**  **n = 3** | ***P*-value** |
| --- | --- | --- | --- |
| Age at ICU admission (years) | 62 (56–69) | 69 (67–71) | 0.303 |
| Male sex | 12 (75) | 2 (67) | >0.999 |
| Bulbar symptoms | 7 (44) | 1 (33) | >0.999 |
| Duration since disease onset (months) | 25 (14–47) | 11 (10–12) | 0.064 |
| ALS diagnosis in ICU | 5 (31) | 2 (67) | 0.607 |
| Intubation in another ICU | 6 (38) | 3 (100) | 0.174 |
| Delay between admission and intubation (days) | 0 (0–1) | 6 (3–9) | 0.471 |
| Indication for intubation |  |  |  |
| Hypoxemia | 6 (38) | 0 (0) | 0.545 |
| Hypercapnia | 8 (50) | 3 (100) | 0.331 |
| Arterial gas at intubation |  |  |  |
| pH | 7.34 (7.25–7.43) | 7.37 (7.36–7.43) | 0.467 |
| PaO_2_ (mmHg) | 81 (67–94) | 46 (46–46) | 0.235 |
| PaCO_2_ (mmHg) | 52 (40–76) | 72 (71–72) | 0.325 |
| Lactate (mmol/L) | 1.1 (0.7–1.3) | - | - |
| HCO3– (mmol/L) | 28 (25–30) | 41 (41–41) | 0.183 |
| Ventilation duration (days) | 12 (5–18) | 7 (6–13) | 0.823 |
| Tracheostomy during ICU stay | 3 (19) | 0 (0) | >0.999 |
| ICU length of stay | 18 (7–34) | 19 (18–30) | 0.576 |
| NIV at discharge | 5 (56) | 3 (100) | 0.480 |
| In–ICU mortality | 7 (44) | 0 (0) | 0.430 |
| In–hospital mortality after ICU discharge | 2 (13) | 0 (0) | >0.999 |

Results are expressed as median (interquartile range) for quantitative variables and as number (percentage) for qualitative variables.

ICU, intensive care unit; PaO_2_, partial pressure of arterial oxygen; PaCO_2_, partial pressure of arterial carbon dioxide

^*^ Out of 12 patients discharged from ICU (9 with hypoxemia and 3 without hypoxemia)

**Table S5. Follow-up outcome after ICU admission, comparison between patients with and without hypoxemia**

|  | **All patients**  **n = 139** | **ARF with hypoxemia**  **n = 82** | **ARF without hypoxemia**  **n = 57** | ***P*-value** |
| --- | --- | --- | --- | --- |
| Death 3 months after ICU admission | 67 (48) | 49 (60) | 18 (32) | **0.002** |
| Rankin score 3 months after ICU admission* | 4 (4–5) | 4 (4–5) | 4 (3–4) | 0.149 |
| Death 6 months after ICU admission | 85 (61) | 59 (72) | 26 (46) | **0.003** |
| Rankin score 6 months after ICU admission^†^ | 4 (4–5) | 4 (4–5) | 4 (4–5) | 0.342 |
| Death 12 months after ICU admission | 103 (74) | 68 (83) | 35 (61) | **0.008** |
| Rankin score 12 months after ICU admission^‡^ | 4 (3–5) | 4 (3–5) | 4 (4–4) | 0.390 |

Results are expressed as median (Q1-Q3) for quantitative variables and as number (percentage) for qualitative variables.

ICU, intensive care unit;

^*^ Out of 69 survivors with available data at 3 months (31 and 38)

^†^ Out of 52 survivors with available data at 6 months (23 and 29)

^‡^ Out of 36 survivors with available data at 12 months (13 and 23)

**Table S6. Sensitivity analysis** on daily non-invasive ventilation use**, factors associated with 3-month mortality, multivariate survival analysis**

| **Variable** | **HR (95% CI)** | ***P*-value** |
| --- | --- | --- |
| Age, *per year increase* | 1.03 (1.00-1.06) | **0.025** |
| Sex, *male* | 1.06 (0.62-1.83) | 0.826 |
| Duration since disease onset, *per year increase* | 1.06 (0.94-1.18) | 0.337 |
| Bulbar symptoms | 0.98 (0.54-1.79) | 0.957 |
| BMI, *per point increase* | 0.97 (0.91-1.03) | 0.308 |
| Hypoxemia at ICU admission | 2.20 (1.24-3.90) | **0.007** |
| Daily non-invasive ventilation use  No  Under 12h per day  Above 12h per day | 1.00 (reference).  1.43 (0.77-2.64)  0.55 (0.19-1.59) | 0.223 |

Hazard ratio (HR) and 95% confidence intervals (CI) computed using multivariate survival analysis

ALS, amyotrophic lateral sclerosis; BMI, body mass index

**Table S7. Sensitivity analysis on ALS diagnosis during the ICU, factors associated with 3-month mortality, multivariate survival analysis**

| **Variable** | **HR (95% CI)** | **p–value** |
| --- | --- | --- |
| Age, *per year increase* | 1.03 (1.00-1.05) | **0.031** |
| Sex, *male* | 1.12 (0.65-1.91) | 0.686 |
| ALS diagnosis during the ICU | 0.44 (0.18-1.05) | 0.063 |
| Bulbar symptoms | 1.06 (0.60-1.87) | 0.833 |
| BMI, *per point increase* | 0.97 (0.91-1.03) | 0.341 |
| Hypoxemia at admission | 2.15 (1.21-3.81) | **0.008** |
| Prior home NIV prescription | 0.97 (0.56-1.67) | 0.900 |

Hazard ratio (HR) and 95% confidence intervals (CI) computed using multivariate survival analysis

ALS, amyotrophic lateral sclerosis; BMI, body mass index
